# The impact of demographic change on the estimated future burden of infectious diseases: examples from hepatitis B and seasonal influenza in the Netherlands

**DOI:** 10.1186/1471-2458-12-1046

**Published:** 2012-12-05

**Authors:** Scott A McDonald, Alies van Lier, Dietrich Plass, Mirjam EE Kretzschmar

**Affiliations:** 1 Centre for Infectious Disease Control, National Institute for Public Health and the Environment, PO Box 1, Bilthoven, 3720, BA, the Netherlands; 2 Department of Public Health Medicine, School of Public Health, University of Bielefeld, Bielefeld, Germany; 3 Julius Centre for Health Sciences & Primary Care, University Medical Centre Utrecht, Utrecht, The Netherlands

**Keywords:** Population ageing, Influenza, Hepatitis B, Disease burden, Disability-adjusted life-years

## Abstract

**Background:**

For accurate estimation of the future burden of communicable diseases, the dynamics of the population at risk – namely population growth and population ageing – need to be taken into account. Accurate burden estimates are necessary for informing policy-makers regarding the planning of vaccination and other control, intervention, and prevention measures. Our aim was to qualitatively explore the impact of population ageing on the estimated future burden of seasonal influenza and hepatitis B virus (HBV) infection in the Netherlands, in the period 2000–2030.

**Methods:**

Population-level disease burden was quantified using the disability-adjusted life years (DALY) measure applied to all health outcomes following acute infection. We used national notification data, pre-defined disease progression models, and a simple model of demographic dynamics to investigate the impact of population ageing on the burden of seasonal influenza and HBV. Scenario analyses were conducted to explore the potential impact of intervention-associated changes in incidence rates.

**Results:**

Including population dynamics resulted in increasing burden over the study period for influenza, whereas a relatively stable future burden was predicted for HBV. For influenza, the increase in DALYs was localised within YLL for the oldest age-groups (55 and older), and for HBV the effect of longer life expectancy in the future was offset by a reduction in incidence in the age-groups most at risk of infection. For both infections, the predicted disease burden was greater than if a static demography was assumed: 1.0 (in 2000) to 2.3-fold (in 2030) higher DALYs for influenza; 1.3 (in 2000) to 1.5-fold (in 2030) higher for HBV.

**Conclusions:**

There are clear, but diverging effects of an ageing population on the estimated disease burden of influenza and HBV in the Netherlands. Replacing static assumptions with a dynamic demographic approach appears essential for deriving realistic burden estimates for informing health policy.

## Background

Advances in medical science and improvements in nutrition, housing, sanitation and other hygenic conditions have contributed towards reducing mortality rates in the Netherlands and globally, with the result of prolonging the average length of life. Life expectancy (LE) in the Netherlands has increased over the period 1950–2010 from 70.3 to 78.8 years for men, and from 72.6 to 82.8 years for women [1]. This rising trend in LE, in combination with fertility rates continuing at replacement level or slightly higher, is predicted to increasingly skew the age distribution of the population towards older ages. According to forecasts by Statistics Netherlands (CBS), in 2050 there will be 4.5 million people in the Netherlands aged 65 years and over [2] – representing 25% of the projected total population – a substantial increase over the 15% of the total population represented by this age category in 2008.

Demographic changes such as population ageing have an impact on estimates of the disease burden (i.e., mortality and morbidity) over time. The immune system becomes less efficient as it ages, with the consequence that for many infectious diseases symptoms can occur more frequently and with greater severity in elderly compared with younger individuals [3]. Thus, when projecting population-based disease burden into the future, the dynamics of demographic processes such as birth, mortality, and migration that influence the age-distribution of the population need to be taken into account.

We investigated the effects of two aspects of demographic change – population ageing and growth – on the future burden of two infectious diseases in the Netherlands. Hepatitis B (HBV) and seasonal influenza were selected as examples because their natural histories differ widely, especially with respect to time-scale. Severe, life-threatening sequelae of HBV develop slowly, often appearing many years following acute infection [4,5], with most of the disease burden attributable to these sequelae. Thus, HBV is a useful infection for illustrating the impact of increased life expectancy predicted for the future time at which sequelae tend to appear. The natural history of seasonal influenza and its complications operates on a much shorter time-scale than HBV, but because both the symptomatic attack rate and the risk of death are strongly age-dependent [6,7], the impact of population ageing on the disease burden attributable to influenza may be substantial. Furthermore, HBV and influenza also differ with respect to the age-groups most at risk of infection; the incidence of HBV is highest in persons in their 30s [8], whereas influenza mainly affects the younger population (5–14 years), with the highest rates for complications observed in <1 years and in 65 years and older [9].

In the current study, we integrated a simple dynamic model of demographic change with established methodology for the computation of disease burden, to assess the impact of population ageing and growth on the disease burden of seasonal influenza and HBV infection in the Netherlands over the period 2000–2030. Our goal was not a high degree of precision, but rather we aimed to show qualitatively the differences in estimated burden when demographic change is taken into account.

## Methods

### DALY methodology

We used the pathogen-based approach for estimating disease burden, in which the infection event serves as the starting point, and all (future) health outcomes – acute infection as well as short and long-term sequelae – causally related to infection with the pathogen are included in the total disease burden calculation [10,11].

We calculated disease burden using the disability-adjusted life-years (DALY) measure, a concept developed by the World Health Organization [12]. As a normative health gap measure, DALYs quantify the difference (in years) between an ideal health situation and the actual health status associated with illness, disease, or injury. One DALY corresponds to one lost year of completely healthy life, or to multiple years lived with disability (i.e., experienced at less than full health). The DALY measure is the simple sum of two components: years of life lost (YLL) due to premature death and years of life lost due to disability (YLD).

An advantage of the DALY methodology is that diseases (both communicable and non-communicable) can easily be compared to each other with respect to their impact on population health. DALY calculation for the pathogen-based disease burden approach requires a number of sources of data: (i) an outcome tree describing disease progression through various health outcomes with specified transition probabilities of developing each outcome, (ii) data on the incidence of infection, disability weights (on a scale from 0 to 1, where 0 represents perfect health and 1 represents death) and the average duration associated with each health outcome, and the rates of mortality from each outcome, and (iii) life expectancy for all ages (typically aggregated into 5-year age-groups).

Incidence data is typically retrieved from national notification or hospitalisation databases, and where necessary is adjusted using multiplication factors (MFs) to correct for under-estimation. Under-estimation can be divided into under-ascertainment (cases at the community level who do not seek healthcare, possibly because infection is mild or even asymptomatic) and under-reporting (cases who have attended healthcare services, but who are not reported to any notification or surveillance system, possibly because notification is not mandatory, notification failure, misdiagnosis or misclassification). YLD and YLL are computed as summations over *j* age groups and *k* health outcomes, including and leading from acute infection at time *t*:

(1a) $$\text{YLD}=\sum_{a=1}^{j}\sum_{i=1}^{k}n_{i,a}d_{i,a}w_{i,a}$$

(1b) $$\text{YLL}=\sum_{a=1}^{j}\sum_{i=1}^{k}m_{i,a}{\text{LE}}_{a}$$

where *n*_*i*,*a*_ is the number of cases developing health outcome *i* among age-group *a*, *d*_*i*,*a*_ and *w*_*i*,*a*_ are the disability duration and disability weight for outcome *i* among age group *a*, *m*_*i*,*a*_ is the number of deaths occurring in health outcome *i* among age group *a*, and *LE*_*a*_ is the life expectancy for age-group *a* at time *t*+*d*_*i*,*a*_.

DALYs were estimated for acute infections occurring during the period 2000–2030, and 95% confidence intervals were constructed around DALYs using Latin hypercube sampling methods [13]. All computations and dynamic modelling were carried out using R statistical software [14].

### Modelling demographic dynamics

Dynamic modelling of the Netherlands population was undertaken also with the year 2000 as a starting point, using demographic information publicly available from CBS for that year (population size according to sex and 1-year age-group (from 0 to 85+ years), age- and sex-specific mortality rates [1], age-specific fertility rates, and age-specific net migration rates [15]). Life expectancy for each age-group was then calculated from mortality rates using standard life-table methods. Evolution of the size and age distribution of the population over the simulation period was computed using Leslie matrices [16], and simple assumptions were implemented with respect to temporal trends in fertility rates (an 0.08% annual increase in fertility rate was assumed for women >27 years) and mortality rates (annual decrease of 1.7% (males) and 1.3% (females), with larger decreases in the oldest age groups). Sex- and age-specific net migration rates were fixed as the average net migration over the period 2000–2009. These modelling assumptions resulted in comparable population sizes and age distributions to actual data available for 2009 and to forecasts by CBS for 2030.

### Modelling disease progression

#### Hepatitis B

The outcome tree for HBV disease progression was adopted from that developed as part of the Burden of Communicable Diseases in Europe (BCoDE) project [10]. This model specifies five health outcomes potentially leading from acute infection: fulminant liver failure, chronic hepatitis, compensated cirrhosis, decompensated cirrhosis, and hepatocellular carcinoma (HCC) (Additional file 1: Figure S1). Transition probabilities, disability weights and durations, and MFs to correct for under-estimation of incident cases were adapted from those compiled for the BCoDE project (see Additional file 1: Table S1 for details).

Acute HBV cases in the Netherlands have been notifiable since 1976 and are openly available; data on age, sex, transmission route, and country of birth are included. In the period 2000–2010 for example, the majority of acute HBV notifications occurred in males (76.3%), and in the 35–39 years age group (15.1%; mean of 38 notified cases per year). Notification counts were first adjusted to account for the under-estimation of the incidence of acute infection when using notification data (range: 2.4–3.0) [17-19]. A second MF was applied to correct for asymptomatic cases not attending health care services (MF: 4.6) [18]. We then calculated mean age- and sex-specific incidence over the period 2000 through 2010, and assumed as a default scenario that incidence rates remained constant over the simulation period (i.e., fixed at the average incidence rate over the period 2000–2010). The age- and sex-aggregated mean corrected incidence rate was 1.9 per 10,000 persons (range: 1.4–2.5). The stratified corrected incidence rates used are provided in Additional file 1: Table S3.

Disease burden, in DALYs per year, was then computed (Eqs. 1a and 1b above) taking into consideration the evolving population age-structure and life expectancy and dynamically calculated incidence. To isolate the effect of demographic change on future disease burden, DALYs were also computed using a ‘static’ demographic model in which the age distribution, population size, and life expectancy were assumed to remain constant throughout the simulation period.

Scenario analyses examined the effect of decreasing incidence rates over the period 2012–2030 (crudely simulating the effects of increasing coverage of HBV vaccination in all age-groups) by 2% per year, and the effect of decreasing incidence rates in the youngest age-groups only (<15 years) of 5% per year (simulating decreases in transmission attributable to age-targetted vaccination [20]).

### Seasonal influenza

The outcome tree for seasonal influenza and most parameter values were also taken from the BCoDE project and earlier work [10,11,21]. The disease progression model specifies three long-term health outcomes leading from acute infection: permanent disability from Acute Respiratory Distress Syndrome, otitis media, or sepsis (Additional file 1: Figure S2). The highest attack rate is seen among young people, with the greatest risk of mortality observed in the elderly [6,7].

We calculated age-specific incidence rates from Dutch sentinel GP consultation rates (data were obtained from the Netherlands Institute for Health Services Research (NIVEL)) for ILI averaged over the period 2000–2010 [22], combined with an estimate of the proportion of ILI cases that were seropositive for influenza (32.2%) [23]. Rates were corrected for under-ascertainment of symptomatic infection (i.e., not going to a GP) using a MF range of 1.96–2.27; this was derived from two studies on the incidence of seasonal influenza [24,25]. The age-aggregated mean corrected incidence rate was 10.9 per 1000 persons (range: 4.9-19.0); the age-stratified corrected incidence rates are given in Additional file 1: Table S3.

The rate of GP consultations due to influenza in the UK (period 1991–1996) has been reported to be 9.9/1000 person-years in the 65+ years age group, 16.4/1000 in the 15–64 years age group, and 12.2/1000 in those aged <15 years [26]. A Dutch study conducted for the period 2002 through 2008 [27] reported average GP consultation rates for influenza-like-illness (ILI) of 19.7 consultations per 1000 children aged 0–4 years (via modelling, the authors attribute 9.3/1000 to infection with the influenza virus), and a rate of 9.7/1000 in 5–15 year olds (attributing 6.3/1000 to influenza). A recent study indicated that the incidence of ILI in the Netherlands is decreasing over time [28].

Over 90% of influenza-related mortality occurs in adults aged 65 years or older [29]. Age-specific mortality rates were taken from a recent Dutch study on influenza-associated mortality [30], which were available for the broad age-groups 0–4, 5–24, 25–44, 45–64, 65–74, and 75+ years. These were converted to case-fatality rates using the corrected age-specific incidence rates derived from ILI data. We computed the disease burden associated with influenza in the presence of population growth and ageing, as done for HBV. For comparison, DALYs were also estimated using a ‘static’ demographic model (i.e., population size, age distribution, and life expectancy were held constant throughout the simulation period).

Influenza vaccine uptake in persons aged 60 years or older has been approximately 75% since 2008 [31]. In order to investigate the potential consequences of more (or less) effective age-targetted vaccination in the future, a scenario analysis was conducted assuming annual decreases in incidence rates in the oldest age groups (60+ years) of 2%, and 5%, and an annual increase of 2% per year, starting in 2012.

## Results

### Population dynamics

**Figure 1:**
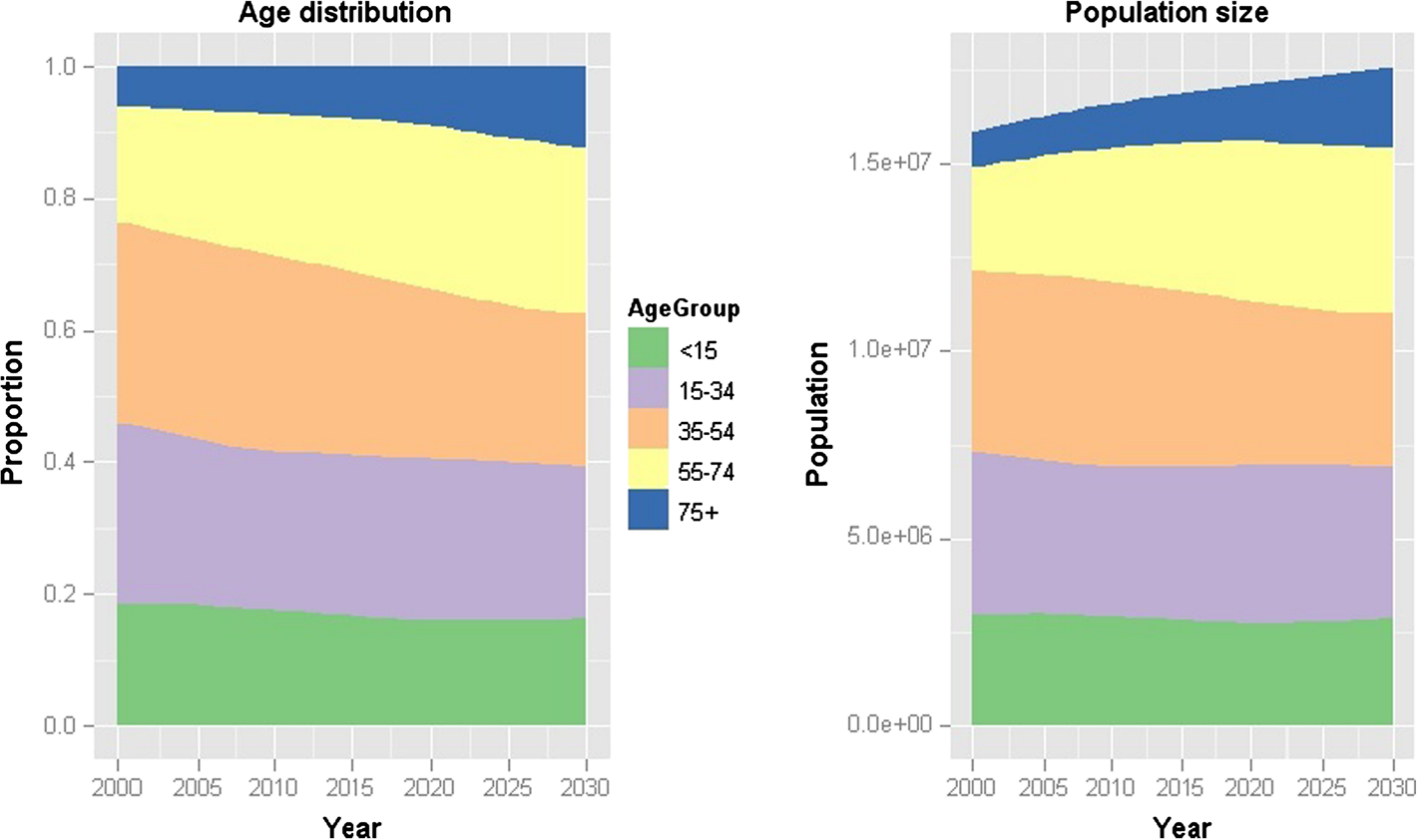
Simulated change in the age distribution of the Netherlands population over the period 2000 to 2030 (left panel) and the corresponding change in the size of each age-group over the same period (right panel).

Figure 1 shows the predicted evolution of the population age distribution as a result of the modelled changes in age-specific mortality and fertility rates over the period 2000–2030. Population ageing is illustrated by a steady increase over time in the proportion of the population in the age groups 55–74 years and 75 years or older, with a corresponding decrease in the proportions for the age-groups under 55 years.

### Hepatitis B

**Figure 2:**
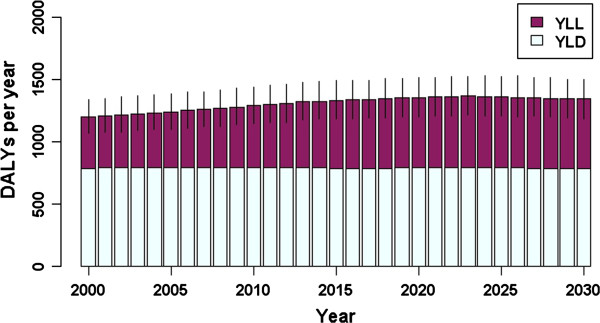
**The modelled annual disease burden of HBV in the Netherlands (split into YLD and YLL per year), for acute infections in the period 2000–2030.** Vertical lines indicate 95% confidence intervals.

**Figure 3:**
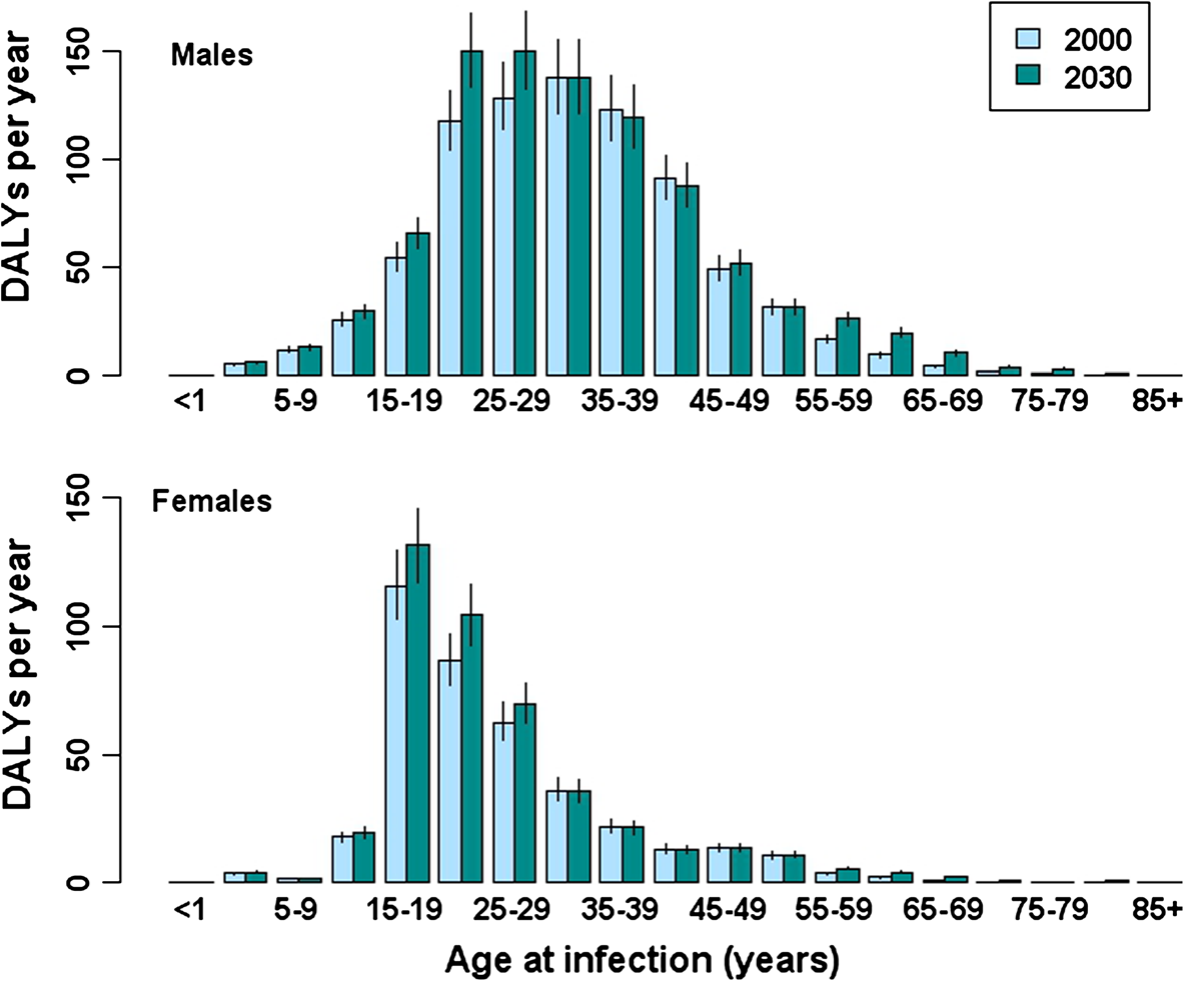
**Age-group specific estimated burden of HBV, comparing acute infections occurring in the years 2000 and 2030, plotted separately for males (upper) and females (lower).** Vertical lines indicate 95% confidence intervals.

**Figure 4:**
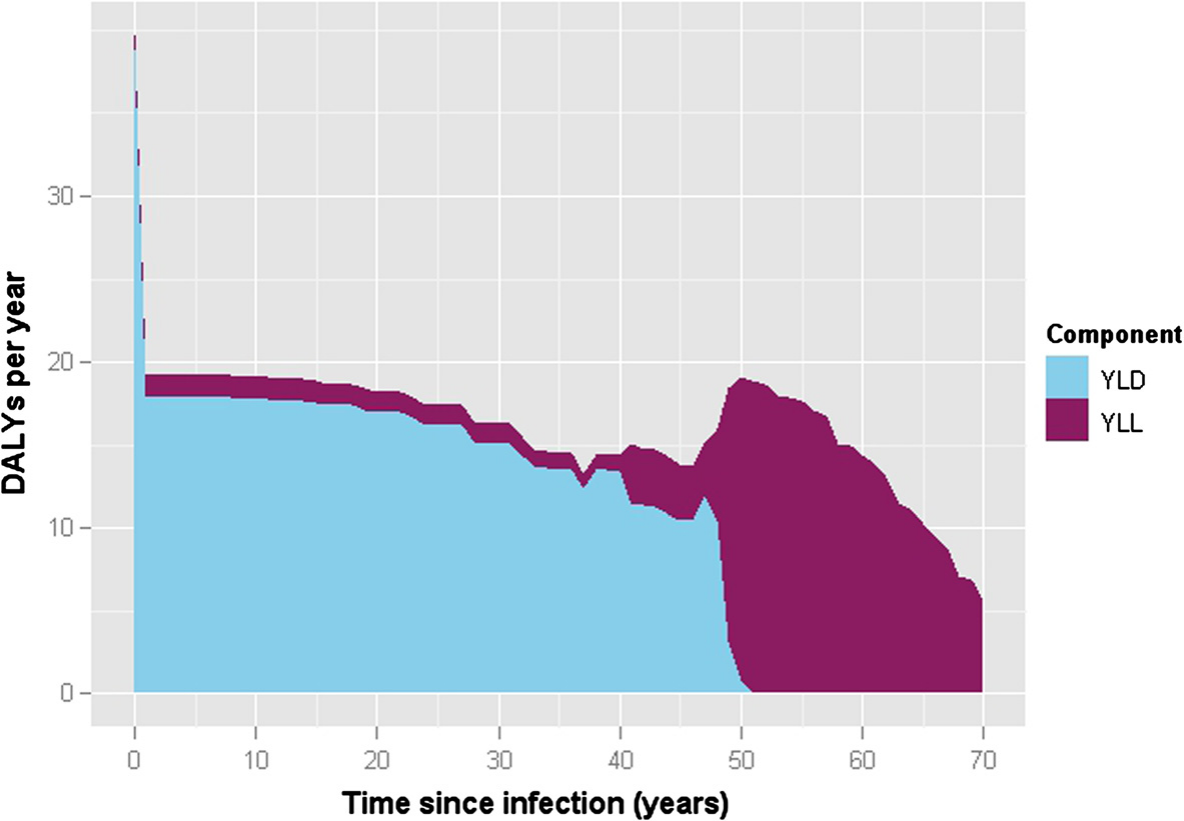
Estimated HBV burden as a function of time since infection, for acute infections occurring in the year 2000 only.

The annual disease burden of HBV in the Netherlands (in DALYs, split into YLD and YLL per year) predicted using the dynamic model is shown in Figure 2. The overall burden increased slightly over the simulation period, from 1196 DALYs (95% CI: 1003–1328; 413 YLL, 95% CI: 352–486) in the year 2000 to 1343 DALYs (95% CI: 1194–1493; 561 YLLs, 95% CI: 483–650) in 2030. Considering infection in the year 2000 only, in males the greatest disease burden of 136 DALYs occurred in the age group with the highest incidence (reflecting the largest number of susceptibles and the highest risk of infection: 30–34 years) (Figure 3), but a relatively high burden is also apparent for the 20–24 years age group, mainly due to high YLL (116 DALYs for males, 86 DALYs for females). Of all age groups, persons aged 20–24 years at infection had the highest YLL, 104, attributable to relatively high incidence and an increased probability of developing severe sequelae at middle age. Consistent with the natural history of HBV-related disease, the majority of the burden is comprised of YLD until approximately 45 years following infection, after which the proportion of YLL increases (Figure 4).

**Figure 5:**
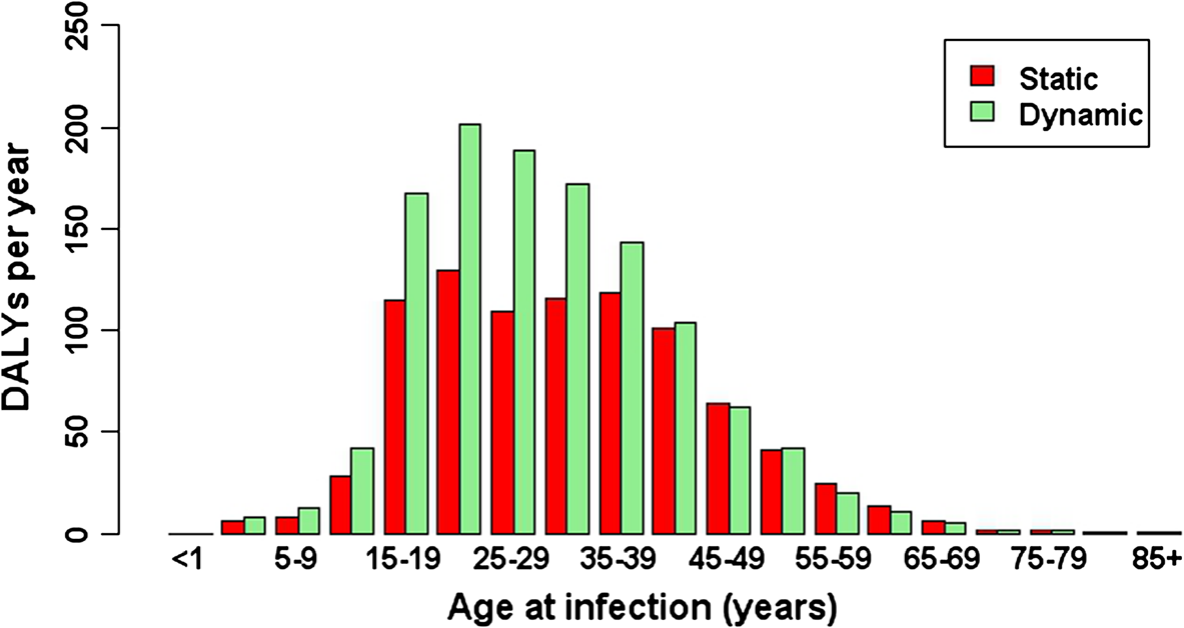
Comparison of dynamic with static modelling assumptions with respect to population growth and ageing on the annual burden of HBV in the Netherlands (in DALYs per year), for acute infections occurring in the year 2000 and aggregated across sex.

Compared with results using a ‘static’ demographic model, in which the population size, age distribution, and life expectancy were held constant, the dynamic model predicted an overall increased burden, ranging from 1.34-fold higher burden in 2000 through a 1.50-fold higher burden in 2030. This greater burden for the dynamic model was largely represented by persons aged 15–39 years at infection (Figure 5).

**Figure 6:**
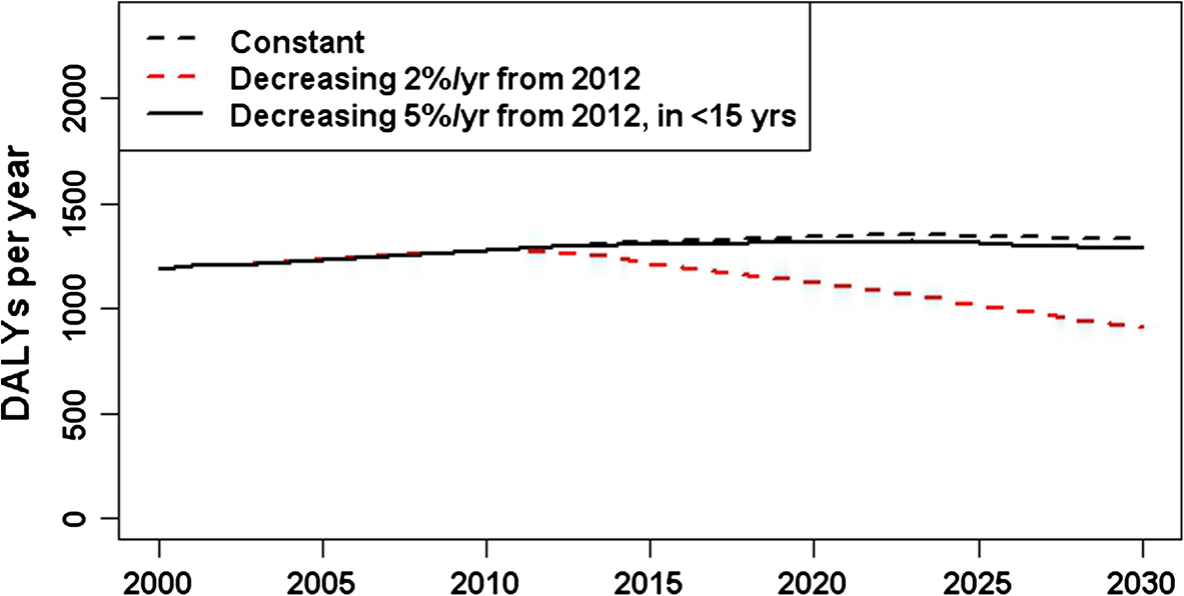
HBV burden estimates in three scenarios: age-specific incidence rates are constant over time; decreasing incidence rates of 2% per year from 2012 (corresponding to increasing vaccination rates in all age groups); and decreased incidence rates of 5% per year in the under-15 years age-groups only (reflecting the effect of age-targetted vaccination).

The scenario analysis indicated a decrease of 32% in the expected DALYs in 2030 if the incidence rate decreased by 2% per year in all age groups from 2012, compared with the constant incidence rate assumption (Figure 6). In the scenario simulating an annual 5% decrease from 2012 in incidence rates in children under 15 years old, the expected total DALYs were 97% of that for the baseline scenario. Approximately 16 acute infections in 2030 were prevented in this scenario.

### Influenza

**Figure 7:**
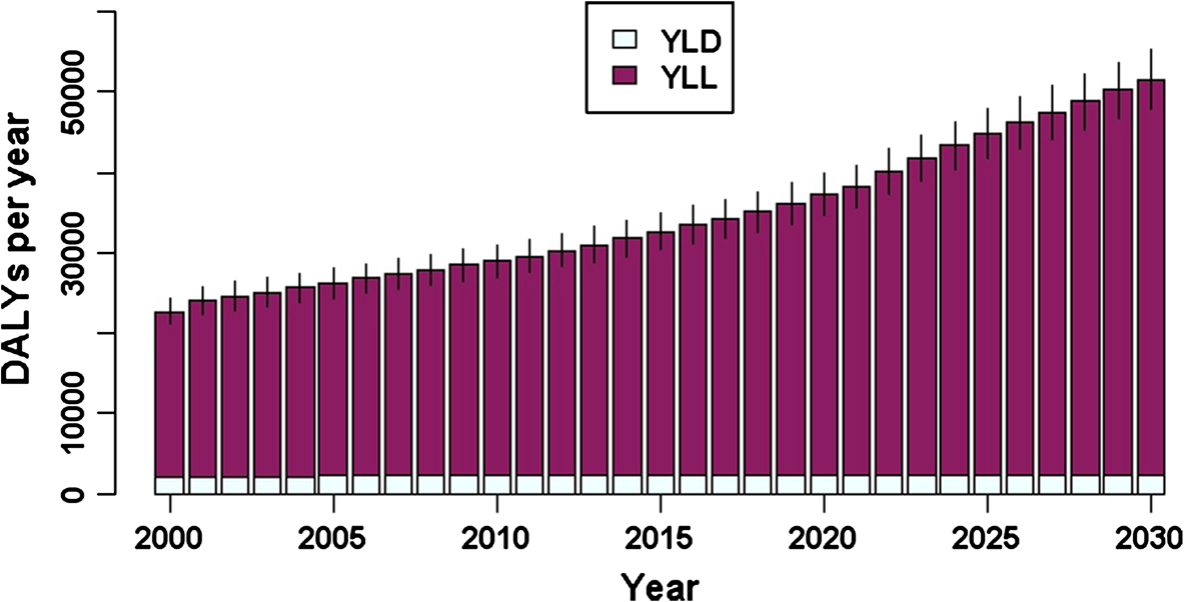
**The modelled annual disease burden of seasonal influenza in the Netherlands (split into YLD and YLL per year), for acute infections occurring in the period 2000–2030.** Vertical lines indicate 95% confidence intervals.

**Figure 8:**
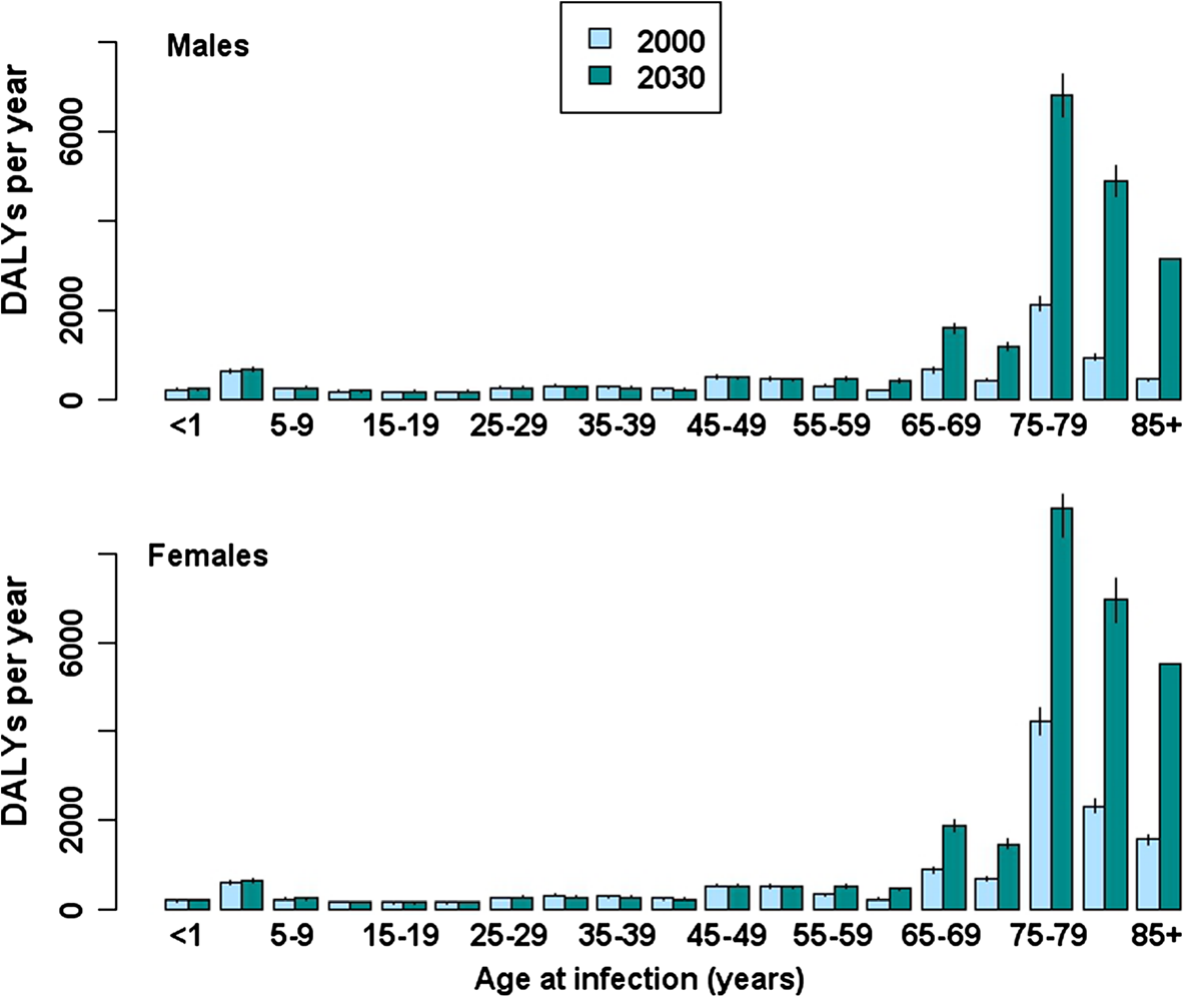
**Age-group specific estimated burden of seasonal influenza, comparing acute infections occurring in the years 2000 and 2030, plotted separately for males (upper) and females (lower).** Vertical lines indicate 95% confidence intervals.

Figure 7 shows the annual disease burden of seasonal influenza. Assuming unchanging incidence rates over time, the total estimated burden increased from 22,712 DALYs (95% CI: 21,132–24,290) associated with infection in 2000, to 51,609 (95% CI: 48,212–55,198) in 2030. The proportion of DALYs comprised by YLL increased from 90.8% to 95.5% across the same time period. There was an increased burden in persons aged 55 or older, with the largest increases predicted for the oldest age groups (75 years and older); the effect of population ageing was also more apparent in females than in males (Figure 8). This increased burden in persons aged 55 and older simultaneously reflects a growing denominator (more elderly people in future) and increasing LE over time (DALYs per infected case in persons 75 years and older increased from 1.08 in 2000 to 1.47 in 2030). The burden for persons aged 25–54 years dropped over the simulation period, following the expected proportional decrease in the size of this age group.

Compared with a ‘static’ population model (with constant population size, age distribution, and life expectancy across the simulation period), the model incorporating demographic dynamics resulted in a total disease burden varying between 1.00-fold (in 2000) and 2.27-fold (in 2030) higher.

**Figure 9:**
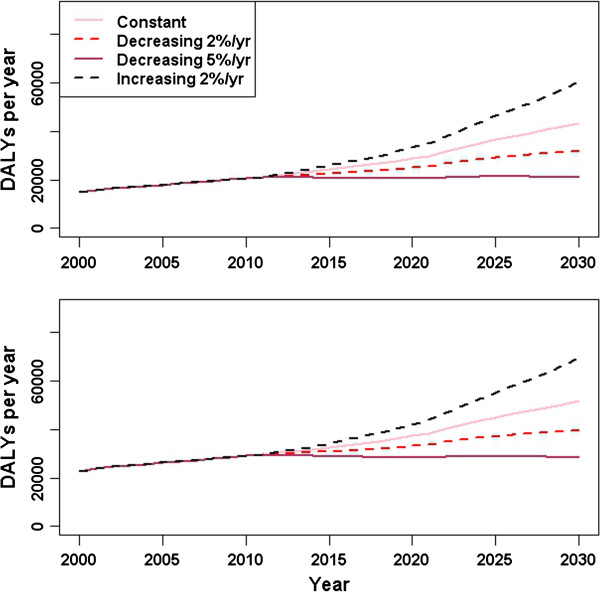
**Influenza burden estimates in four scenarios for trends in incidence rates from 2012, for the 60+ years age group only (upper) and for all ages combined (lower).** In each scenario, the affected incidence rates for the 60+ age group were (i) constant, (ii) decreased by 2% per year from 2012, (iii) decreased by 5% per year, and (iv) increased by 2% per year.

The scenario analysis investigating the consequences of decreasing trends in incidence rates for the 60+ years age group from 2012 indicated substantial reductions in the overall disease burden, mainly in YLL, were achieved with moderate reductions in the incidence of infection (Figure 9). For the scenarios assuming 2% and 5% decreasing incidence rate trends, the expected total DALYs for influenza infection in 2030 were 77% and 55%, respectively, of the expected total DALYs in the baseline scenario of constant incidence rates. If incidence rates in this age group increased at 2% per year, then the expected total DALYs in 2030 were 134% that of the baseline scenario.

## Discussion

By simulating the ageing and growth of the Dutch population over the period 2000 through 2030, we found diverging trends in the future disease burden associated with infection with acute HBV and seasonal influenza. For both infections considered, incorporation of a simple model of population growth and ageing predicted a higher overall disease burden for both infections than if population size, age distribution, and life expectancy were assumed to remain constant over time (the steady-state assumption). For HBV, this greater disease burden is mainly attributable to the increased life expectancy at the time sequelae develop; note that this effect is larger for HBV (total DALYs for persons infected in 2000 were 1.3 times higher; Figure 5) compared with influenza. The difference is in part due to the very different time-scales of progression to sequelae of the two diseases. For influenza, the effect of population ageing is more pronounced for infection occurring in the future, when the elderly segment of the population – for which both incidence and mortality rates are high – has substantially increased. DALYs were estimated at 2.3 times higher than predicted by the static model for infection with influenza in the year 2030.

For HBV, population ageing also meant that the relative size of the population in the age group most at risk for acute infection diminished over the simulation period, giving rise to fewer incident infections overall and a lower projected future burden. At the same time, the increase in life expectancy over the simulation period predicts an increase in the opportunity to develop long-term sequelae, and for those persons who die from HBV-related severe sequelae, the YLL component of the disease burden will also increase (Figure 2). These two opposing influences on the burden resulted in a relatively stable trend in DALYs over the simulation period.

In contrast, for seasonal influenza our results indicated an overall increasing burden over the simulation period. The effect of population ageing is mostly attributable to the growing size of the elderly population. This effect is localised in YLL, due to increased life expectancy over time and the highest mortality rates occurring in the oldest age groups. The effect of population ageing on disease burden was more pronounced for the female than the male elderly, due to longer female life expectancy.

We note that our estimated influenza burden is much higher than previously reported for seasonal influenza [11] or for the 2009 H1N1 pandemic [21]. These previous studies computed DALYs based on reported influenza mortality, whereas we incorporated modelled mortality rates [30] to avoid underestimation due to limited laboratory confirmation or influenza not being reported as the primary cause of death.

The evolution of the age distribution in the population is entirely driven by the demographic processes of mortality, fertility, and migration. We have not attempted to closely fit population-level data on temporal trends in these rates; our goal was to show the impact on disease burden from a typical ageing population that is the consequence of such processes; which does not require accurate model fit.

Previous research has used models of transmission dynamics to predict the effects of demographic change (population decline and ageing) on the incidence of childhood disease [32,33]. Other work has employed projections for cause-specific mortality rates and population growth/ageing to estimate the future global burden of disease (e.g., the burden in 2030 based on WHO 2002 estimates) in terms of DALYs [34], but has not specifically examined the impact of population ageing. Using a simple extrapolation method, a Dutch study estimated the relative change in the burden attributable to nine infectious agents in 2050, based on prevalence/incidence in 2008 and projections for the population age-group distribution [35]. Our study is the first to investigate the effects of ageing on disease burden using the DALY methodology and realistic models of disease progression.

One limitation of the current study is that we have assumed that the parameters underlying transmission dynamics do not change over time; incidence rates reflect the force of infection (determined by contact patterns between age/risk groups and the prevalence of acute infection in the population), and interventions such as vaccination will influence future incidence. In addition, demographic changes such as population growth and ageing can influence the transmission of infection through shifts in the size of the subpopulations that are susceptible to infection. Our findings thus illustrate the simple scenario in which transmission dynamics remain constant, localising any trends in disease burden to population dynamics only.

A second limitation concerns data quality, namely the assumptions underlying the estimation of annual incidence. For HBV, a single MF was applied over the entire simulation period to account for under-estimation inherent in the notification data, namely under-reporting and under-ascertainment of cases. For influenza, the MF range was based on two studies only (conducted in Hong Kong in 2008 and Scotland in 1993–1994); thus, generalisability to the Netherlands setting is an issue. The accuracy of the calculated DALYs depends strongly on the accuracy of the reconstructed incidence of acute infection, and thus also on the MFs.

For HBV we have not considered the influence of acute infection in first-generation migrants (FGMs) from endemic countries, or secondary transmission from FGMs. Based on serological survey data from 2007, it is estimated that 1.4% of FGMs in the Netherlands are chronically infected, representing 51% of the infected population [36]. Screening and treatment of this high-risk group has been shown to be cost-effective in preventing future HBV-related burden [37]. As a consequence of improved screening/treatment of FGMs from high-prevalence countries, higher vaccination coverage of their children, and/or prevention initiatives in the countries of origin, the incidence rate for acute infection may decrease over time, reducing the anticipated future disease burden.

Furthermore, for HBV we have simplified certain parameters; e.g., we assumed the disability durations for compensated cirrhosis and HCC to be equivalent for males and females and constant for all ages. For influenza, age-specific disability weights for the long-term sequela of otitis media were simplified to a single value.

Finally, the pathogen-based approach attributes all future burden arising from initial infection to the year of infection. Figure 4 indicates the temporal aspects of the HBV disease burden, by projecting disease progression over time; it can be seen for acute infections in 2000, a step increase approximately 45 years after infection is apparent, with the majority burden component changing from YLD to YLL. Such information on the temporal variation in the burden of diseases with long natural histories may be useful for the strategic planning of adequate medical care and the provision of timely intervention measures.

## Conclusions

The ageing of the population is expected to have a clear impact on the future disease burden of HBV and influenza in the Netherlands; computing disease burden using steady-state assumptions is not realistic. We emphasise that our results should not be interpreted as forecasts, but rather as a qualitative investigation into the effects of population change. As such, results are generalisable to other countries with similar demographic dynamics and age-specific patterns in incidence. Future work is required to refine these findings; for instance, by incorporating the time-dependent effects of potential public health interventions, changes in contact patterns, risk factors, or, in the case of HBV, the number of chronic carriers entering the population [36], as well as evolutionary changes in the pathogen; all can influence transmission, and thus the incidence of infection and future burden of disease.

## Abbreviations

BCoDE: Burden of Communicable Diseases in Europe; CI: Confidence interval; CBS: Statistics Netherlands; DALY: Disability-adjusted life-years; HBV: Hepatitis B virus; MF: Multiplication factor; ILI: Influenza-like illness; YLD: Years of life lost due to disability; YLL: Years of life lost.

## Competing interests

The authors declare that they have no competing interests.

## Authors’ contributions

SM conceptualised the study, carried out the simulations and burden computations, and drafted the manuscript. MK conceptualised the study, advised on modelling, interpreted the results, and edited the manuscript. AvL and DP interpreted the results and edited the manuscript. All authors read and approved the final manuscript.

## Pre-publication history

The pre-publication history for this paper can be accessed here:

http://www.biomedcentral.com/1471-2458/12/1046/prepub

## Supplementary Material

Additional file 1 Outcome trees and parameter values for hepatitis B and influenza. Click here for file
