# Supplementary material for: The impact of demographic change on the estimated future burden of infectious diseases: examples from hepatitis B and seasonal influenza in the Netherlands
Source: BMC Public Health. 2012 Dec 5;12:1046. doi: 10.1186/1471-2458-12-1046 (PMC3537516; doi:10.1186/1471-2458-12-1046)
Supplement: Additional file 1 — Outcome trees and parameter values for hepatitis B and influenza. [file 1471-2458-12-1046-S1.pdf]

Additional file 1

The impact of demographic change on the estimated future burden of infectious diseases: Examples from hepatitis B and seasonal influenza in the Netherlands

Scott A. McDonald, Alies van Lier, Dietrich Plass, Mirjam E. Kretzschmar

(BMC Public Health. Submitted 2012)

Figure A1. Outcome tree for hepatitis B

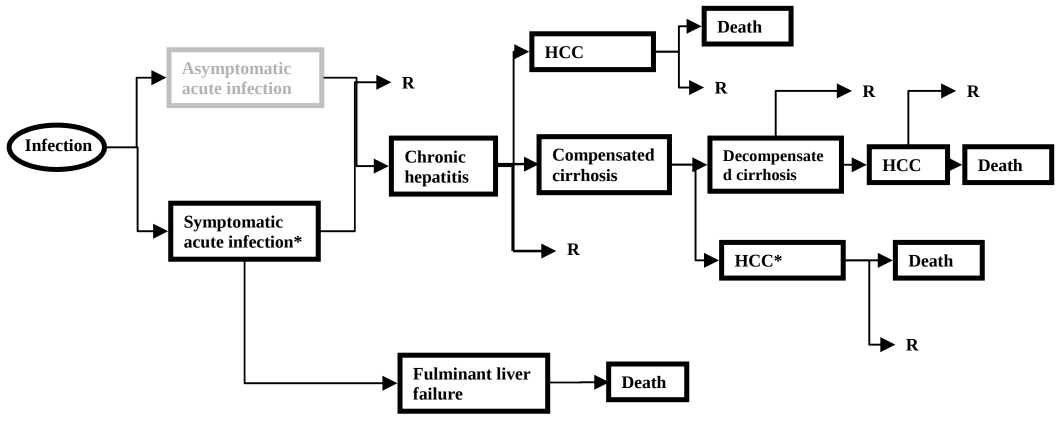

Note. R = recovered; HCC = hepatocellalar carcinoma

Table A1. Hepatitis B disease progression probabilities and other parameters[10].

**Health outcome**	**Transitional probability**	**Disability weight**	**Duration (years)**
Acute hepatitis	--	0.211	0.17
Fulminant liver failure	0.5–1%	0.809	0.0918
Chronic hepatitis	0.001–0.01%	0.0958	37.736
Compensated cirrhosis	2.1%/yr	0.33	9.0
Decompensated cirrhosis	5–7%/yr	0.809	1.429
Hepatocellular carcinoma, following:		0.809	2.8
Chronic hepatitis	0.1–1%/yr		
Comp. cirrhosis	3%/yr		
Decomp. cirrhosis	3%/yr		
Death, following:		--	--
Fulminant liver failure	20–33.3%		
Decomp. cirrhosis	57–77%/yr		
Hepat. carcinoma	49%/yr		

Figure A2. Outcome tree for seasonal influenza

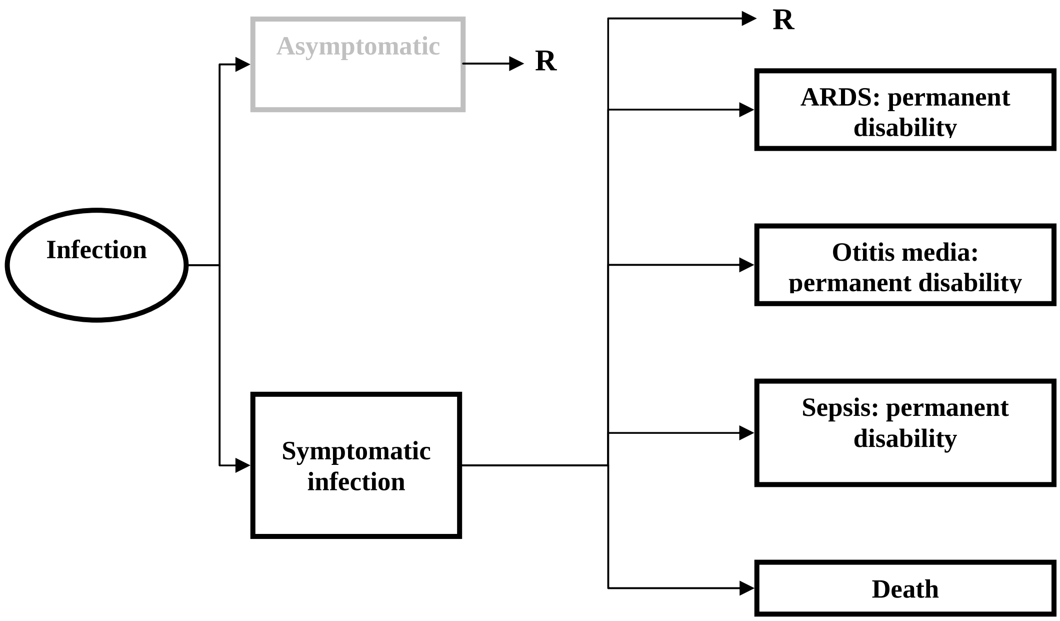

Table A2. Influenza disease progression probabilities and other parameters.[10]

Health outcome	Transitional probability	Disability weight	Duration
Symptomatic infection<br>*Acute Respiratory Distress Syndrome (ARDS) (0.02%)*<br>*Otitis media (0.65%)*<br>*Sepsis (0.01%)*	–	0.01*	2 weeks
Permanent disability due to *ARDS*	0.000112	0.18	Remaining life expectancy
Permanent disability due to *Otitis media (deafness)*	0.00000065	0.17	Remaining life expectancy
Permanent disability due to *Sepsis*	0.000082	0.28	Remaining life expectancy
Death, following acute infection (per 10,000)	6.69 (0-4 yrs)<br>3.74 (5-24 yrs)<br>6.90 (25-44 yrs)<br>24.8 (45-64 yrs)<br>140 (65-74 yrs)<br>1260 (75+ yrs)	--	--

* Annual profile disability weight. Years lived with disability (YLD) was computed using the annual profile method for disability weight determination (focusing on the year in which an episode of acute illness is experienced).

Table A3. Estimated age-specific incidence rates (per 10,000 persons per year) for hepatitis B and seasonal influenza in the Netherlands.

Age group	HBV (males)	HBV (females)	Influenza
<1 year	0	0	323
0-4	0.084	0.059	242
5-9	0.18	0.024	119
10-14	0.52	0.33	93.8
15-19	1.5	2.8	94.0
20-24	4.0	2.6	93.5
25-29	4.6	2.0	88.9
30-34	5.6	1.3	101
35-39	6.2	1.0	104
40-44	5.8	0.77	102
45-49	4.1	0.96	93.7
50-54	3.0	0.90	98.5
55-59	2.5	0.48	98.9
60-64	2.0	0.38	95.6
65-69	1.3	0.26	94.4
70-74	0.63	0.11	101
75-79	0.75	0.081	104
80-84	0.29	0.22	119
85+	0	0.16	124
